# Circular RNA CircFAM188B Encodes a Protein That Regulates Proliferation and Differentiation of Chicken Skeletal Muscle Satellite Cells

**DOI:** 10.3389/fcell.2020.522588

**Published:** 2020-11-06

**Authors:** Huadong Yin, Xiaoxu Shen, Jing Zhao, Xinao Cao, Haorong He, Shunshun Han, Yuqi Chen, Can Cui, Yuanhang Wei, Yan Wang, Diyan Li, Qing Zhu

**Affiliations:** Farm Animal Genetic Resources Exploration and Innovation Key Laboratory of Sichuan Province, Sichuan Agricultural University, Chengdu, China

**Keywords:** circFAM188B, protein coding, SMSCs, proliferation, differentiation

## Abstract

Circular RNAs (circRNAs) are recognized as functional non-coding transcripts; however, emerging evidence has revealed that some synthetic circRNAs generate functional peptides or proteins. Additionally, the diverse biological functions of circRNAs include acting as miRNA-binding sponges, RNA-binding protein regulators, and protein translation templates. Previously, we found that circular RNA circFAM188B is a stable circular RNA and differentially expressed between broiler chickens and layers during embryonic skeletal muscle development. In this study, we found that circFAM188B exhibited a unique pattern of sharply decreased expression from embryonic day 10 (E10) to Day 35 (D35) after hatching. Our experimental results showed that circFAM188B promotes the proliferation, but inhibits the differentiation of chicken skeletal muscle satellite cells (SMSCs). Bioinformatic analysis revealed circFAM188B contain an opening reading frame (ORF) which translate into circFAM188B-103aa, internal ribosome entry site (IRES) analysis further confirmed the coding potential of circFAM188B. In addition, western blot assay detected a flag tagged circFAM188B-103aa, and several peptides of circFAM188B-103aa were detected by LC-MS/MS analysis. We further verified that the role of circFAM188B-103aa in chicken myogenesis is consistent with that of its parent transcript circFAM188B, which facilitates proliferation, but represses differentiation of chicken SMSC. Taken together, these results suggested that a novel protein circFAM188B-103aa encoded by circFAM188B that promotes the proliferation but inhibits the differentiation of chicken SMSCs.

## Introduction

With the development of RNA deep-sequencing technology and bioinformatics during the last few years, human and animal genomes in general have been shown to be transcribed into many different and complex RNA families (David et al., 2014). Additionally, a diverse collection of new antisense, intronic and intergenic transcripts have been identified with alternative transcriptional start sites, differences in termination and splicing patterns, and non-coding RNAs representing nearly half of the total RNAs (Cesana et al., 2011). Circular RNAs (circRNAs) are a special subtype of non-coding RNAs with covalently closed continuous loop structure and without 5′ to 3′ polarity, which confers a higher tolerance to exonucleases (Rybakwolf and Al, 2015). Due to their conservation, abundance and specificity, circRNAs perform diverse biological functions, including acting as miRNA-binding sponges, RNA-binding protein regulators, and protein translation templates (Li X. et al., 2018).

Traditionally, it has been accepted that non-coding RNAs cannot be translated into proteins due to the absence of the hallmark ORFs longer than 100 codons (Li et al., 2017); however, emerging evidence has revealed that functional peptides or proteins can be generated from some so-called “non-coding RNAs,” including long non-coding RNAs (lncRNAs), primary miRNAs (pri-miRNAs), and synthetic circRNAs (Yang et al., 2018). Although circRNAs have been shown to be translated in principle, Pamudurti et al. provided the first direct evidence that circMbl is generated from the *Mbl* Locus, and produces a detectable endogenous protein in Drosophila (Pamudurti et al., 2017). Subsequently, circ-ZNF609 was shown to be associated with heavy polysomes, and translated into a protein in a splicing-dependent and cap-independent manner in murine and human myoblasts (Legnini et al., 2017). Then, a few translatable circRNAs were identified play a significant role in cancer cell development, such as circFBXW7, circSHPRH, circAKT3 and so on (Shi et al., 2020).

Skeletal muscle is the most abundant striated muscle tissue, accounting for 40–60% of adult animal body weight, plays a vital role in initiating movement, maintains homeostasis, and supports respiration (Zacharewicz et al., 2013). Additionally, loss of skeletal muscle function and mass is associated with a variety of diseases, including cancer and diabetes (Gea et al., 2012). Chicken is a well-established model of skeletal muscle formation in vertebrates, as the developmental anatomy of chicken skeletal muscles is very similar to that of mammals (Federica et al., 2015).

In our previous study, we sequenced the circRNAs of 24 embryonic chicken breast muscles and identified 228 circRNAs that were differentially expressed between broilers and layers (Shen et al., 2019). CircFAM188B was identified as one of the differentially expressed circRNAs detected at high levels (ranking of expression level: 25/4,226) during chicken embryonic skeletal muscle development. Next, we confirmed that circFAM188B is a stable circular RNA and expressed at lower levels in broilers than in layers at embryonic day 10 (E10), E16 and E19 (Supplementary Figure S1). Moreover, we found that circFAM188B contains an ORF. Based on this information, we speculated that circFAM188B influences chicken skeletal muscle development and may be translated into a functional protein.

In this study, we investigated the ability of circFAM188B to regulate the proliferation and differentiation of chicken SMSCs and the underlying mechanism.

## Materials and Methods

### Sample Collection

The use of animal samples in this study was approved by the Animal Care and Use Committee of Sichuan Agricultural University (China). A total of 300 ROSS-308 broiler fertilized egg were obtained from the Sichuan Yuguan Agriculture Co., Ltd. (Sichuan, China). All eggs were incubated in an Automatic Incubator (Oscilla, Shandong, China) at 37.8°C, with 60 ± 10% humidity. We collected breast muscle and leg muscle from three chicks at each time-point from E10 to E20 embryonic age and Day 1 (D1), D3, D5, D7, D14, D21, D28, and D35 after hatching. In addition, we also collected heart, liver, spleen, lung, kidney, breast muscle, leg muscle, brain, intestine, and adipose of three ROSS-308 broiler chickens. All samples collected were immediately frozen in liquid nitrogen and stored at −80°C prior to RNA isolation.

### Vector Construction and RNA Oligonucleotides

The linear sequence of circFAM188B was cloned into pCD2.1-ciR (Geneseed Biotech, Guangzhou, China) using the *Kpn*I and *Bam*HI (Takara, Dalian, China) restriction sites (OV-circFAM188B); the empty vector was used as negative control (OV-NC). The linear sequence of circFAM188B, which includes the sequence encoding Flag tags after the start codon “ATG” (no. 24), was cloned into pCD2.1-ciR, which was designated OV-circFAM188B-Flag. Besides, the start codon “ATG” (no. 24) of the OV-circFAM188B-Flag plasmid was mutated to “CCG” in the OV-circFAM188B-Flag-MT plasmid. Moreover, the linear sequence of flag tagged circFAM188B-103aa was integrated into the *Hin*dIII/*Kpn*I restriction sites of the pcDNA3.1 overexpression plasmid (OV-circFAM188B-103aa); the pcDNA3.1 empty vector was used as the corresponding negative control (empty vector). The IRES-like sequence of circFAM188B was cloned into the Luc2-IRES-Reporter plasmid (Geneseed Biotech) using the *Kpn*I and *Eco*RI (Takara) restriction sites. All sequences were chemically synthesized at Sangon Biotech Co., Ltd. (Shanghai, China). Small interfering RNAs (siRNAs) overlapping circFAM188B junction sites (Supplementary Table S1) were synthesized by GenePharma Co., Ltd. (Shanghai, China).

### Cell Culture and Treatment

Chicken skeletal muscle satellite cells (SMSCs) were isolated from the breast muscles of 4-day-old ROSS-308 chickens. Breast muscles were collected, and after removing skin and bones, the muscles were minced and digested sequentially with 0.2% collagenase Type II (Gibco, Langley, OK, United States) and 0.25% trypsin (Gibco). Digestion reactions were terminated by adding equal values of 10% growth medium [GM: Dulbecco’s modified Eagle’s medium (DMEM) (Gibco) + 10% fetal bovine serum (Gibco) + 0.2% penicillin/streptomycin (Invitrogen, Carlsbad, CA, United States)]. The suspension was filtered through a cell strainer (pore size, 0.075 mm) and the cells were then isolated by centrifugation. The cells were resuspended in GM and cultured at 37°C in a 5% CO_2_, humidified atmosphere. Serial plating was performed to enrich SMSCs and to remove fibroblasts. GM was replaced every day and differentiation medium [DM: DMEM + 2% horse serum (Gibco)] was used to induce SMSC differentiation. DF-1 cells were cultured in 10% GM, and medium was also replaced every 24 h.

At approximately 90% confluence, SMSCs were transfected using Lipofectamine 3000 (Invitrogen) according to the manufacturer’s instructions. Cells were then cultured in DM for differentiation studies, while cell proliferation was studied in cells transfected at 50% confluence and cultured in GM.

### RNA Isolation, cDNA Synthesis, and Quantitative Real-Time PCR (qRT-PCR)

Total RNA was isolated using TRIzol reagent (Takara) according to the manufacturer’s instructions. cDNA synthesis was performed using TransScript One-Step gDNA Removal and cDNA Synthesis SuperMix (TransGen, Beijing, China) according to the manufacturer’s instructions. The qRT-PCR analyses were performed on three biological replicates at each time-point using TB Green PCR Master Mix (Takara). The β*-actin* gene was used as internal control. The primers are listed in Supplementary Table S2. The 2^–ΔΔCt^ method was used to analyze the relative expression level of qRT-PCR data.

### Nuclear-Cytoplasmic Fractionation

The nuclear and cytoplasmic fractionation assays were performed using NE-PER^TM^ Nuclear and Cytoplasmic Extraction Reagents (Thermo Fisher, Carlsbad, CA, United States) according to the manufacturer’s instructions. Finally, the nuclear and cytoplasmic samples were mixed with 1 mL TRIzol prior to RNA isolation.

### Fluorescence *In situ* Hybridization (FISH) Assay

The FISH probe was designed to span the splice junction of circFAM188B (Supplementary Table S1) and synthesized at GenePharma Co., Ltd. The FISH assays were performed using the Fluorescence *In Situ* Hybridization Kit (GenePharma) according to the manufacturer’s instructions. The images were obtained using a laser scanning confocal microscope (Thermo Fisher).

### Luciferase Reporter Assay

For luciferase reporter assays, DF-1 cells were seeded in 48-well plates and transfected with Luc2-IRES-Reporter vectors as described in section “Vector construction and RNA oligonucleotides”. After for 48 h, the luminescent values of firefly and Renilla luciferases were detected using the LucPair^TM^ Duo-Luciferase Assay Kit (GeneCopoeia, Rockville, MD, United States) with a fluorescence/multi-detection microplate reader (US BioTek Laboratories, Shoreline, WA, United States).

### EdU Assay

EdU assays were performed with Cell-Light EdU Apollo567 *In Vitro* Kits (RiboBio, Guangzhou, China) according to the manufacturer’s protocol. Briefly, SMSCs were seeded in 48-well plates and transfected with siRNAs or vectors for 48 h as described in section “Vector construction and RNA oligonucleotides”. Subsequently, cells were incubated with 50 μM EdU reagent at 37°C for 2 h and the cell nuclei were counter-stained with Hoechst 33342 for 30 min. A fluorescence microscope (Olympus, Japan) was used to capture three randomly selected fields. The number of EdU-stained cells was counted by Image-Pro Plus software.

### Cell Counting Kit 8 (CCK-8) Assay

Chicken skeletal muscle satellite cells were seeded in 96-well plates and transfected with siRNAs or vectors as described in section “Vector construction and RNA oligonucleotides”. After 12 h, 24 h, 36 h, and 48 h, cell proliferation was monitored using a Cell Counting Kit-8 kit (Multi Sciences, Hangzhou, China) according to the manufacturer’s protocol. The absorbance of each sample at a wavelength of 450 nm was measured using a Microplate Reader (Thermo Fisher).

### Flow Cytometric Cell Cycle Analysis

SMSCs were seeded in 12-well plates and transfected with siRNAs or vectors as described in section “Vector construction and RNA oligonucleotides”. After 48 h, cells were collected and suspended in 75% ethanol overnight at −20°C. The cells were then collected and incubated with 500 μl PI/RNase Staining Buffer Solution (BD Biosciences, Franklin Lakes, NJ, United States) at 37°C for 15 min. Flow cytometric analysis was performed using a BD AccuriC6 flow cytometer (BD Biosciences) and Modfit software.

### Immunoprecipitation (IP)

DF-1 cells were seeded in a T25 cell culture flask and transfected with OV-circFAM188B-Flag vector as described in section “Vector construction and RNA oligonucleotides”. After 72 h, the transfected cells were lysed in IP lysis buffer (Thermo Fisher). The lysates were then gently centrifuged and cleared by incubation with 25 μl of protein A/G agarose (Thermo Fisher) for 1.5 h at 4°C. The pre-cleared supernatant was subjected to IP using the indicated primary antibodies at 4°C overnight. The protein complexes were then collected by incubation with 30 μl of protein A/G gel for 2 h at 4°C. The collected protein complexes were separated by sodium dodecyl sulfate-polyacrylamide gel electrophoresis (SDS-PAGE) and analyzed by LC-MS/MS.

### LC-MS Analysis

After separation by SDS-PAGE, and the protein bands were excised from the gel manually and digested with sequencing-grade trypsin (Promega, Madison, WI, United States). The digested peptides were analyzed with a Q Exactive mass spectrometer (Thermo Fisher). The fragment spectra were analyzed using the National Center for Biotechnology Information non-redundant protein database with Mascot (Matrix Science).

### Western Blot Assay

Chicken skeletal muscle satellite cells were seeded in 6-well plates, cultured in DM and transfected as described in section “Vector construction and RNA oligonucleotides”. After 72 h, the proteins of transfected cells were extracted using lysis buffer and the concentration determined using a bicinchoninic acid (BCA) protein assay kit (Beyotime, Shanghai, China). Total proteins (10 μg) were separated by 12% SDS-PAGE, and transferred to a 0.2 mm polyvinylidene fluoride (PVDF) membrane that was soaked in formaldehyde. The membrane was then blocked with blocking Buffer (Beyotime) for approximately 2 h at room temperature before incubation overnight at 4°C with primary detection antibodies [anti-myosin heavy chain (MyHC) (Santa Cruz, CA, United States; 1:1,000) and anti-β-tubulin (ZenBio, Chengdu, China; 1:2,000)]. The PVDF membrane was washed three times with western wash Buffer (Beyotime) and then incubated for 2 h at room temperature with the horseradish peroxidase (HRP)-conjugated secondary detection antibody [anti-mouse immunoglobulin G (IgG) (ZenBio; 1:2,000)]. Finally, antibody reactive bands were detected using enhanced chemiluminescence (ECL) (Beyotime). The relative expression of protein was measured by Image J software.

### Immunofluorescence Assay

Chicken skeletal muscle satellite cells were seeded in 24-well plates, cultured in DM and transfected as described in section “Vector construction and RNA oligonucleotides”. After 72 h, cells were fixed in 4% formaldehyde for 30 min then washed three times with PBS for 5 min. Subsequently, the cells were permeabilized by adding 0.1% Triton X-100 for 20 min and blocked with 5% goat serum (Beyotime) for 30 min. After incubation with anti-MyHC (Santa Cruz; 1:250) at 4°C for 12 h, the Rhodamine (TRITC) AffiniPure Goat Anti-Mouse IgG (ZenBio; 1:1000) was added and the cells were incubated at 37°C for 1 h. The cell nuclei were stained with DAPI (Beyotime; 1:50) for 5 min. Three images were obtained at random using a fluorescence microscope (Olympus, Japan). The area of myotubes was measured by Image-Pro Plus software.

### Statistical Analysis

Data are presented as least squares means ± standard error of the mean (SEM). For two group comparison analysis, statistical significance of differences between the means was analyzed by unpaired Student’s *t*-test and marked by symbol “*”. For multiple group comparison analysis, data were analyzed by one-way ANOVA and marked by letters “a, b, c, d, e and f”. All analysis was performed using SPSS 20.0 software (SPSS Inc., United States). *P* < 0.05 was considered to indicate statistical significance.

## Results

### CircFAM188B Expression Patterns in Chicken

**FIGURE 1:**
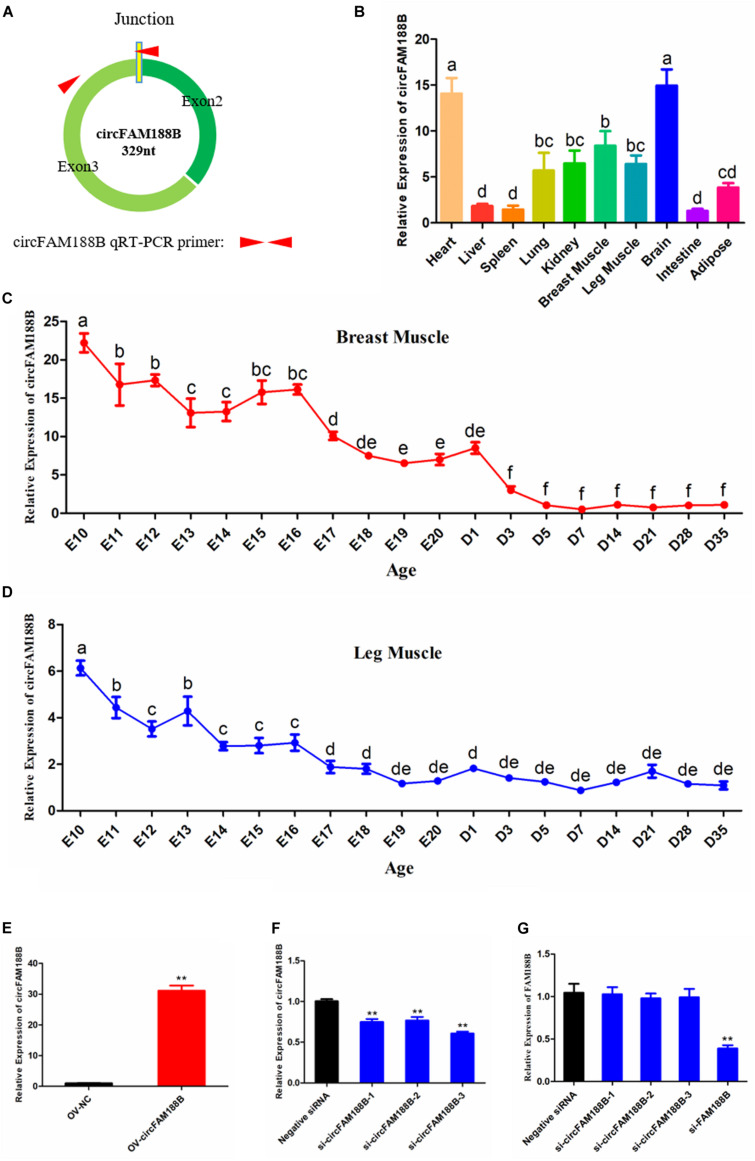
CircFAM188B expression patterns in chicken. **(A)** Schematic diagram of circFAM188B formation and qRT-PCR primer design. **(B)** circFAM188B expression profiles in ROSS-308 chicken tissues. **(C)** The relative RNA level of circFAM188B in the breast muscles of E10-D35 chicken. **(D)** The relative RNA level of circFAM188B in the leg muscles of E10-D35 chicken. **(E)** The relative expression of circFAM188B in SMSCs transfected with OV-circFAM188B or OV-NC vector. **(F)** The relative expression of circFAM188B in SMSCs transfected with circFAM188B-specific siRNA or negative control siRNA. **(G)** The relative expression of FAM188B gene in SMSCs transfected with circFAM188B-specific siRNAs, FAM188B-specific siRNA or negative control siRNA. The results of all groups are shown as mean ± S.E.M., and the data represent three independent assessment methods. The one-way ANOVA **(B–D)** and Student’s *t*-test **(E–G)** were used to compare expression levels among different groups. Different letters means indicate statistical significance. *P < 0.05; **P < 0.01; ^a,b^*P* < 0.05.

The expression pattern of circFAM188B in chicken skeletal muscle development was investigated by qRT-PCR using primer pairs designed with one primer spanning the splice junction (Figure 1A). Our analysis showed that circFAM188B was enriched in heart and brain, and also highly expressed in breast muscle and leg muscle (Figure 1B). We also analyzed the expression of circFAM188B in chicken breast muscle and leg muscle from E10 to D35. In breast muscle, circFAM188B levels decreased sharply from E10 to D35, although the circFAM188B expression levels were much higher during the embryonic period (E10–E20) than those after birth (D1–D35) (*P* < 0.05; Figure 1C). Similarly, in leg muscle, circFAM188B expression levels decreased sharply from E10 to E16, and were then maintained at low levels from E17 to D35 (*P* < 0.05; Figure 1D). Besides, the expression of FAM188B mRNA were also detected by qRT-PCR and results showed a differentially expression pattern from circFAM188B (Supplementary Figure S2).

To investigate the role of circFAM188B in SMSC proliferation and differentiation, cells were transfected with an overexpression vector or siRNAs. Compared with the negative control group, 30-fold greater circFAM188B expression was detected in cells transfected with the overexpression vector (*P* < 0.01; Figure 1E). Conversely, all three siRNAs significantly knocked down the level of circFAM188B relative to that of the siRNA NC group (*P* < 0.01; Figure 1F). Among the three siRNAs, si-circFAM188B-3 had the highest interference efficiency and was chosen for use in subsequent experiments (designated si-circFAM188B). In addition, these siRNAs could not affect the expression of FAM188B mRNA, while the siRNA targeted FAM188B mRNA showed a significantly knockdown effect (Figure 1G).

### CircFAM188B Promotes the Proliferation of Chicken SMSCs

**FIGURE 2:**
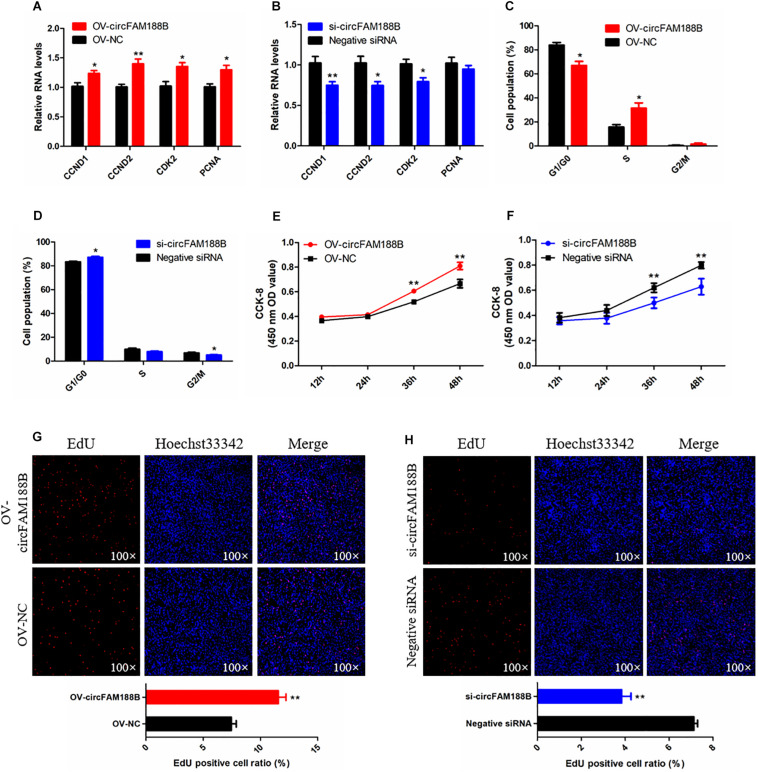
CircFAM188B promotes proliferation of chicken SMSCs. **(A,B)** The relative RNA level of cell proliferation-related genes in chicken SMSCs following circFAM188B overexpression or inhibition. **(C,D)** Cell cycle analysis of SMSCs following circFAM188B overexpression or inhibition. **(E,F)** The growth curve of SMSCs following circFAM188B overexpression and inhibition. **(G,H**) Fluorescence microscope images of proliferating chicken SMSCs labeled with EdU following circFAM188B overexpression or inhibition **(upper panels)**. Proliferation rates of chicken SMSCs following circFAM188B overexpression or inhibition **(lower panels)**. The results of all groups are shown as mean ± S.E.M., and the data represent three independent assessment methods. Student’s *t*-test was used to compare expression levels or values among different groups. *P < 0.05; **P < 0.01.

The effects of circFAM188B on SMSC proliferation were investigated by qRT-PCR, CCK-8, flow cytometric, and EdU assays. qRT-PCR analysis showed that circFAM188B overexpression significantly increased the levels of the cell proliferation-related genes cyclin D1 (*CCND1*), cyclin D2 (*CCND2*), cyclin-dependent-kinase 2 (*CDK2*), and proliferating cell nuclear antigen (*PCNA*) (*P* < 0.05; Figure 2A), whereas expression levels of these genes were decreased by circFAM188B knockdown (*P* < 0.05; Figure 2B). the protein level of CCND1 and PCNA showed a consistent change with their mRNA expression after circFAM188B overexpression or inhibition (Supplementary Figure S3A). Besides, cell cycle analysis showed that circFAM188B overexpression promoted cell cycle progression of SMSCs into the S and G2 phases (*P* < 0.05; Figure 2C and Supplementary Figure S3B), whereas circFAM188B knockdown cell arrest in the G1 phase (*P* < 0.05; Figure 2D and Supplementary Figure S3B). In addition, CCK-8 assay results showed that SMSC proliferation was significantly increased following circFAM188B overexpression (*P* < 0.01; Figure 2E) but significantly decreased following circFAM188B knockdown (*P* < 0.01; Figure 2F). Moreover, the results of the EdU assays were consistent with those of the CCK-8 assays, with significantly reduced SMSC proliferation observed following circFAM188B overexpression (*P* < 0.01; Figure 2G), whereas circFAM188B knockdown significantly increased the population of proliferating cells (*P* < 0.01; Figure 2H). Together, these results suggested that circFAM188B promotes SMSC proliferation.

### CircFAM188B Inhibits Chicken SMSC Differentiation

**FIGURE 3:**
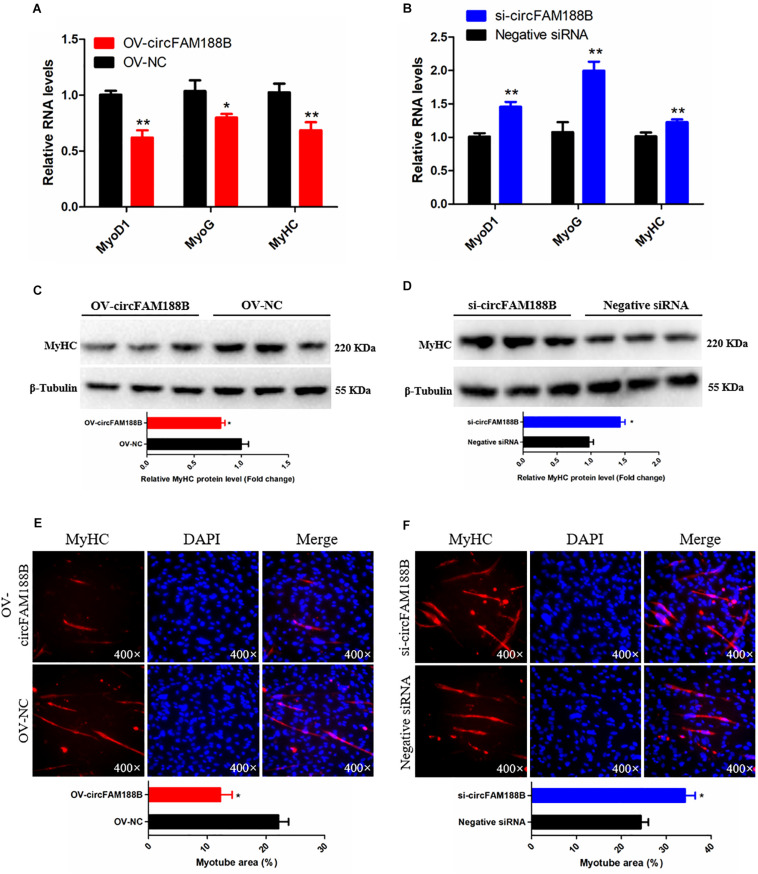
CircFAM188B inhibits chicken SMSC differentiation. **(A,B)** The relative RNA level of muscle cell differentiation marker genes in chicken SMSCs following circFAM188B overexpression or inhibition. **(C,D)** The levels of MyHC and β-tubulin proteins in SMSCs following circFAM188B overexpression or inhibition **(upper panels)**. MyHC band intensities quantified by Image J and normalized against β-tubulin **(lower panels)**. **(E,F)** Images of MyHC staining of SMSCs following circFAM188B overexpression or inhibition **(upper panels)**. Relative myotube area (ratio of MyHC fluorescence area to DAPI area) of SMSCs following circFAM188B overexpression or inhibition **(lower panels)**. The results of all groups are shown as mean ± S.E.M., and the data represent three independent assessment methods. Student’s *t*-test was used to compare expression levels or values among different groups. *P < 0.05; **P < 0.01.

The ability of circFAM188B to regulate SMSC differentiation was investigated by qRT-PCR, western blot and immunofluorescence assays. The qRT-PCR analysis showed that the expression of three muscle differentiation marker genes, myoblast determination protein 1 (*MyoD1*), myogenin (*MyoG*), and *MyHC* were significantly decreased following circFAM188B overexpression (*P* < 0.05; Figure 3A). In contrast, circFAM188B knockdown significantly increased the expression of these muscle differentiation marker genes (*P* < 0.01; Figure 3B). Similarly, MyHC protein levels decreased following circFAM188B overexpression (*P* < 0.05; Figure 3C) but increased when circFAM188B was knocked down (*P* < 0.05; Figure 3D). Moreover, immunofluorescence analysis of MyHC expression showed that the relative area of myotubes was significantly decreased following circFAM188B overexpression (*P* < 0.05; Figure 3E), whereas circFAM188B knockdown increased the relative area of myotubes compared with that in the negative control group (*P* < 0.05; Figure 3F). These results suggested that circFAM188B inhibited SMSC differentiation.

### CircFAM188B Is Located in the Cytoplasm With Coding Potential

**FIGURE 4:**
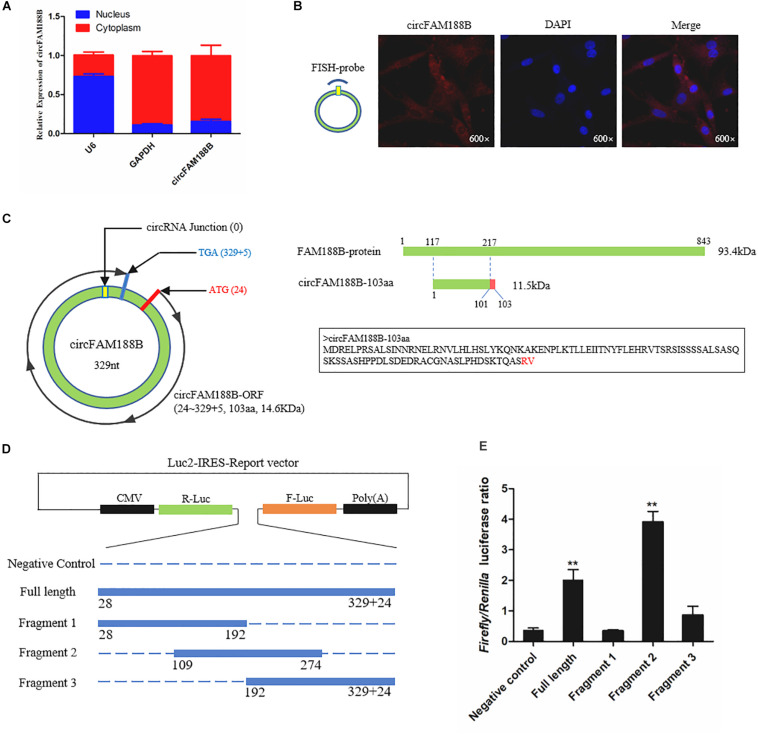
CircFAM188B is located in the cytoplasm with coding potential. **(A)** CircFAM188B expression levels in the cytoplasm (red) and nuclei (blue) of undifferentiated SMSCs. **(B)** FISH assay of circFAM188B in chicken SMSCs. **(C)** Schematic diagram of circFAM188B-103aa. **(D)** Schematic diagram of the dual-luciferase reporter (Luc2-IRES-Reports vector) containing different fragments of circFAM188B, which was used for IRES verification. **(E)** Ratio of Firefly luciferase to Renilla luciferase activity of DF-1 cells after transfection with dual-luciferase reporter vectors shown in **(D)**. The results of all groups are shown as mean ± S.E.M., and the data represent three independent assessment methods. Student’s *t*-test was used to compare expression levels or values among different groups. *P < 0.05; **P < 0.01.

To determine the mechanism by which circFAM188B regulates SMSC proliferation and differentiation, we first confirmed the subcellular localization of circFAM188B. Nuclear-cytoplasmic fractionation assays showed that circFAM188B was present in the cytoplasm of SMSCs (Figure 4A). FISH assays also confirmed the cytoplasmic localization of circFAM188B (Figure 4B). Most previous studies have focused on the ability of circRNAs to regulate biological processes by acting as competing endogenous RNAs to miRNAs (Wei et al., 2017; Chen et al., 2018, 2019; Li et al., 2018a,b, 2019; Ouyang et al., 2018a,b; Peng et al., 2019; Shen et al., 2019; Wang et al., 2019). Therefore, we first used RNAhybrid software to predict the potential miRNA binding sites of circFAM188B, although the results indicated that circFAM188B does not combine with the miRNAs with known functions in muscle development (Supplementary Table S3). On the other hand, we found that circFAM188B contains a non-conservative ORF encoding a protein of 103 amino acids (designated circFAM188B-103aa) with a predicted molecular weight is 11.5 kDa. The predicted protein circFAM188B-103aa shares most of its aa sequence with that of FAM188B, with the exception of two unique amino acids “RV” in the ORF located at the C-terminus (Figure 4C).

First, to demonstrate that circFAM188B contains a natural IRES, which is required for translation initiation in 5′-cap-independent coding RNAs, RNA secondary structure analysis was performed (Supplementary Figure S4), and then we carried out an IRES activity test using the Luc2-IRES-Reporter vector. As shown in Figure 4D, different fragments of the circFAM188B sequence were cloned into the Luc2-IRES-Reporter vector between Ranilla Luciferase (R-Luc) and Firefly Luciferase(F-Luc) sequences. All vectors were transfected into the DF-1 cells, and the luciferase activity of F-Luc relative to that of R-Luc was measured in each group. The results showed that full-length circFAM188B significantly increased the F-Luc luciferase activity, which suggested that circFAM188B contains an IRES-like sequence. Furthermore, fragment 2 of these three fragments from circFAM188B exhibited the greatest effect in promoting F-Luc luciferase activity, which implicated the sequence from 109 to 274 (fragment 2 of circFAM188B) as the potential IRES element of circFAM188B (*P* < 0.01; Figure 4E).

### CircFAM188B Encodes Novel Protein CircFAM188B-103aa

**FIGURE 5:**
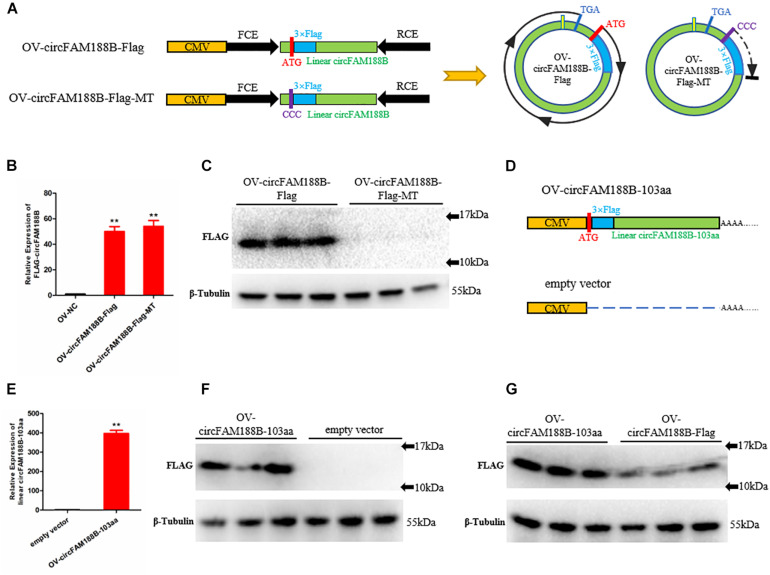
CircFAM188B encodes novel protein circFAM188B-103aa. **(A)** Flag-tagged linear circFAM188B sequence and mutation type sequence (ATG mutation to CCC) were cloned into the pCD2.1-ciR vector **(left)**. Schematic diagram of the ORF of Flag-tagged circular RNA circFAM188B generated from the two linearized vectors shown on the left (right). **(B)** Relative expression of Flag-tagged circFAM188B in SMSCs transfected with Flag-tag vectors. **(C)** Flag-tagged protein levels in SMSCs transfected with Flag-tag vectors. **(D)** The linear Flag-tagged circFAM188B-103aa sequence was cloned into the pCDNA3.1 vector; empty vector was used as a negative control. **(E)** The relative RNA level of linear Flag-tagged circFAM188B-103aa in SMSCs transfected with these Flag-tagged vectors. **(F)** The Flag-tagged protein level of SMSCs transfected with these Flag-tagged vectors. **(G)** Flag-tagged protein levels in SMSCs transfected with OV-circFAM188B-Flag and OV-circFAM188B-103aa vector. The results of all groups are shown as mean ± S.E.M., and the data represent three independent assessment methods. Student’s *t*-test was used to compare expression levels or values among different groups. *P < 0.05; **P < 0.01.

To identify the potential circFAM188B-encoded protein, we inserted a Flag-tagged sequence after the start codon “ATG” in the OV-circFAM188B vector to generate the construct designated OV-circFAM188B-Flag, which theoretically encodes a protein with a Flag-tag (14.6 kDa). In the negative control plasmid, designated OV-circFAM188B-Flag-MT, the start codon “ATG” was mutation to “CCC” (Figure 5A). Transfection of SMSCs with the OV-circFAM188B-Flag vector and OV-circFAM188B-Flag-MT vector both successfully resulted in the overexpression of circFAM188B (*P* < 0.01; Figure 5B). Western blot analysis confirmed the presence of a Flag-tagged protein (molecular weight 10–17 KDa) in the OV-circFAM188B-Flag transfected group, but not in the OV-circFAM188B-Flag-MT group (Figure 5C). On the other hand, we constructed another circFAM188B-103aa overexpression vector, OV-circFAM188B-103aa. This construct contained the linear sequence of Flag-tagged circFAM188B-103aa cloned into pCDNA3.1 plasmid. The empty vector pCDNA3.1 served as a corresponding negative control (Figure 5D). Following transfection of SMSCs, qRT-PCR analysis showed that OV-circFAM188B-103aa vector resulted in 400-fold overexpression of linear circFAM188B-103aa compared with the level detected in the cells transfected with the negative control (*P* < 0.01; Figure 5E). western blot results also indicated a distinct Flag-tagged protein band appeared in the OV-circFAM188B-103aa transfected group (Figure 5F). Importantly, OV-circFAM188B-Flag and OV-circFAM188B-103aa vector generated similar size protein with different efficiency (Figure 5G). Taken together, these results provide preliminary evidence that circFAM188B can be translated into a protein.

### Identification of Novel Protein CircFAM188B-103aa

**FIGURE 6:**
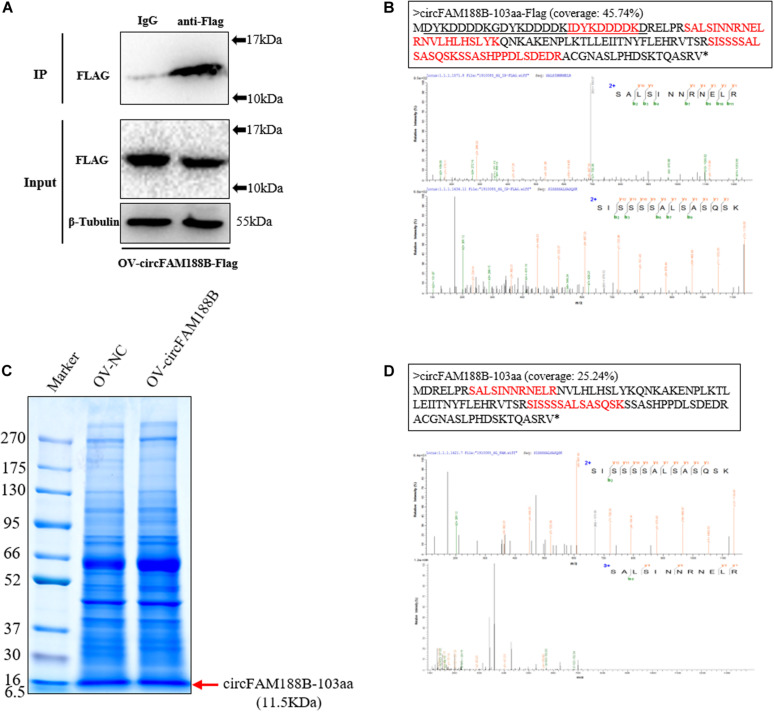
Identification of the novel protein circFAM188B-103aa. **(A)** The Flag-tagged protein of the sample used in IP assays was detected by western blot analysis. **(B)** LC-MS/MS-identified amino acid sequences of the Flag-tagged circFAM188B-103aa (red, **upper panels**), the specific charge of corresponding peptide **(lower panels)**. **(C)** Total cell lysates were separated by SDS-PAGE. Bands representing proteins between 10–17 kDa were excised manually and submitted for identification by LC-MS/MS. **(D)** LC-MS/MS-identified amino acid sequences of the circFAM188B-103aa (red, **upper panels**), the specific charge of corresponding peptide **(lower panels)**.

To obtain evidence of the novel circFAM188B-encoded protein, circFAM188B-103aa, we transfected OV-circFAM188B-Flag into DF-1 cells. We then performed immunoprecipitation assays on DF-1 cells lysates using an anti-Flag antibody (Figure 6A), and then isolated proteins of 10–17 kDa by SDS-PAGE. In LC-MS/MS analysis of these proteins, we identified five amino acid sequences (IDYKDDDDK, SALSINNRNELR, NVLHLHSLYK, SISSSSALSASQSK, and SSASHPPDLSDEDR) of the Flag-tagged circFAM188B-103aa protein (Figure 6B and Supplementary Figure S5). To directly confirm that circFAM188B-103aa was translated from circFAM188B, we then transfected OV-circFAM188B vector into SMSCs and isolated proteins of 6.5–16 KDa in size (Figure 6C). LC-MS/MS analysis revealed two peptides (SALSINNRNELR and SISSSSALSASQSK) that matched the circFAM188B-103aa sequence (Figure 6D).

### CircFAM188B-103aa Promotes the Proliferation of Chicken SMSCs

**FIGURE 7:**
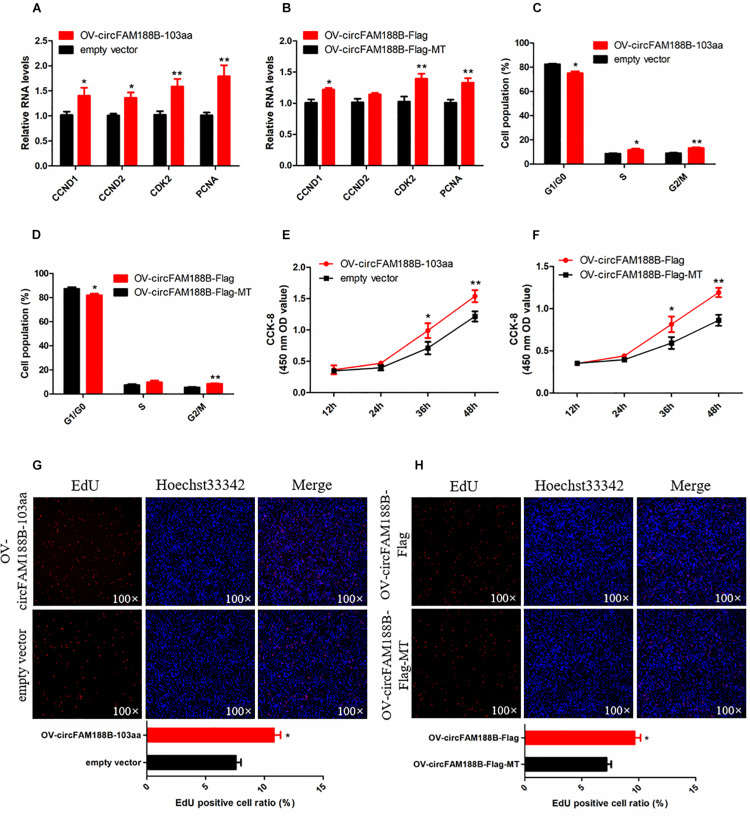
CircFAM188B-103aa promotes proliferation of chicken SMSCs. **(A,B)** The relative RNA level of cell proliferation-related genes in chicken SMSCs following circFAM188B-103aa overexpression. **(C,D)** Cell cycle analysis of SMSCs following circFAM188B-103aa overexpression. **(E,F)** The growth curve of SMSCs following circFAM188B-103aa overexpression. **(G,H)** Fluorescence microscope images of proliferating chicken SMSCs labeled with EdU following circFAM188B-103aa overexpression **(upper panels)**. Proliferation rates of chicken SMSCs following circFAM188B-103aa overexpression **(lower panels)**. The results of all groups are shown as mean ± S.E.M., and the data represent three independent assessment methods. Student’s *t*-test were used to compare expression levels or values among different groups. *P < 0.05; **P < 0.01.

To explore the effect of circFAM188B-103aa on SMSC proliferation, we transfected the four vectors mentioned in section “CircFAM188B encodes novel protein circFAM188B-103aa” (Figures 5A,D) into SMSCs. Our qRT-PCR analysis showed both the OV-circFAM188B-Flag and OV-circFAM188B-103aa vectors promoted the mRNA and protein expression of cell proliferation-related genes in SMSCs (*P* < 0.05; Figures 7A,B and Supplementary Figure S6A). Moreover, cell cycle analysis showed that circFAM188B-103aa overexpression promoted the progression of SMSCs from the G1 phase into the S and G2 phases (*P* < 0.05; Figures 7C,D and Supplementary Figure S6B). In addition, CCK-8 assays showed that SMSC proliferation was significantly increased following transfection with the circFAM188B-103aa overexpression vectors (*P* < 0.05; Figures 7E,F). The CCK-8 assay results were confirmed in EdU assays, which showed that circFAM188B-103aa overexpression significantly increased the ratio of proliferating cells (*P* < 0.05; Figures 7G,H).

### CircFAM188B-103aa Inhibits Chicken SMSCs Differentiation

**FIGURE 8:**
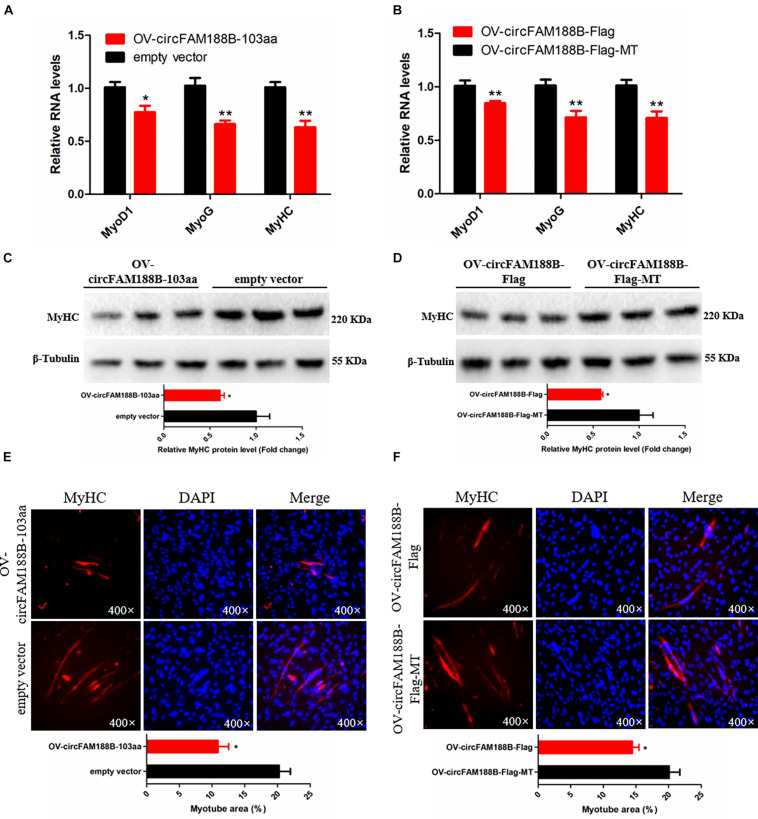
CircFAM188B-103aa inhibits chicken SMSC differentiation. **(A,B)** The relative RNA level of muscle cell differentiation marker genes in chicken SMSCs following circFAM188B-103aa overexpression. **(C,D)** The levels of MyHC and β-tubulin proteins in SMSCs following circFAM188B-103aa overexpression **(upper panel)**. MyHC band intensities were quantified using Image J and normalized against β-tubulin **(lower panels)**. **(E,F)** Image of MyHC staining of SMSCs following circFAM188B-103aa overexpression **(upper panels)**. Relative myotube area (ratio of MyHC fluorescence area to DAPI area) of SMSCs following circFAM188B-103aa overexpression **(lower panel)**. The results of all groups are shown as mean ± S.E.M., and the data represent three independent assessment methods. Student’s *t*-test was used to compare expression levels or values among different groups. *P < 0.05; **P < 0.01.

To investigate the role of circFAM188-103aa in SMSC differentiation, the four different vectors generated in this study (Figures 5A,D) were transfected into SMSCs. The qRT-PCR analysis showed that circFAM188B-103aa overexpression significantly decreased the RNA levels of three muscle cell differentiation-related genes (*MyoD1*, *MyoG*, and *MyHC*) (*P* < 0.05; Figures 8A,B). Similarly, western blot assays showed that MyHC protein levels were decreased by circFAM188B-103aa overexpression (*P* < 0.05; Figures 8C,D). Furthermore, MyHC immunofluorescence analysis demonstrated that circFAM188B-103aa overexpression decreased the relative myotube area of SMSCs (*P* < 0.05; Figures 8E,F).

## Discussion

With the development of genome research techniques, it has been shown that transcription of the animal genome is not only ubiquitous, but also incredibly complex. Recently, circRNAs have come under scrutiny due to its distinct circular structure. Accumulating evidence shows that circRNAs play important roles in many biological processes, including skeletal muscle development (Das et al., 2019). In our previous study, RNA-seq analysis of breast muscles obtained from embryonic broilers and layers revealed 228 differentially expressed circRNAs. After experimental verification, we confirmed higher expression of a novel circular RNA, circFAM188B, in the breast muscle of layer chickens compared with that of broilers at E10, E16 and E19 (Shen et al., 2019). In the present study, we found that circFAM188B expression peaked in the early embryonic period (E10–E16), followed by the late embryonic period (E17–D1), with the lowest expression detected after birth (D3–D35) both in breast muscle and leg muscle. The circFAM188B expression pattern indicates that it may be involved in skeletal muscle development.

Next, we carried out a series of experiments to illuminate the role of circFAM188B in chicken skeletal muscle development. Our results showed that circFAM188B promotes the proliferation of chicken SMSCs, but play a negative role in SMSCs differentiation. Previous reports have revealed several circRNAs play roles in chicken myogenesis, circSVIL promotes myoblasts differentiation and proliferation by sponging miR-203 (Ouyang et al., 2018a). CircHIPK3 also play a positive role in myoblasts proliferation and differentiation (Chen et al., 2019). Interestingly, circSVIL and circHIPK3 exhibited a different expression pattern with circFAM188B, which suggest the unique function association with their own expression profiles.

Recent studies of circRNAs have indicated that the “miRNA sponge” function represents the most conspicuous mechanism of their effects, although the general roles of circRNAs remain to be fully elucidated. Interestingly, current evidence shows that circRNAs can be translated in eukaryotic cells (Chen and Sarnow, 1995; Legnini et al., 2017; Pamudurti et al., 2017). However, evidence of circular RNA encoded proteins has been found only in viruses (Kos et al., 1986; AbouHaidar et al., 2014), bacteria (Perriman and Ares, 1998), Drosophila (Pamudurti et al., 2017), and humans (Abe et al., 2015; Yang et al., 2017; Zhang et al., 2018a,b; Zheng et al., 2019). Translatable circRNAs are defined a class of 5′-cap-independent coding RNAs based on their distinct structure. The IRES is a vital element for cap-independent coding RNA translation (Komar et al., 2012). In addition, a recent study showed that N6-methyladenosine (m6A) also facilitates circRNA translation (Yang et al., 2017).

In the present study, we showed that circFAM188B is localized in the cytoplasm and contains an ORF. Moreover, IRES tests revealed the existence of a potential IRES sequence at position 109–274 of the linear sequence of circFAM188B, which suggested that circFAM188B has the potential to encode a protein. Then, we constructed a series of Flag-tag vectors to assess circFAM188B translation. Indeed, a novel Flag-tagged protein was detected in cells transfected with the wild-type OV-circFAM188B-Flag vector construct, but not in the cells transfected with the construct in which the start codon “ATG” was mutated to “CCC.” In addition, a new vector named OV-circFAM188B-103aa, could transcribe a linear RNA which produce a same size flagged protein as OV-circFAM188B-Flag. These results indicated that circFAM188B can be translated. Furthermore, IP assays were performed to enrich the Flag-tagged protein. LC-MS/MS analysis to identify the circFAM188B translated protein revealed several peptides matching the protein translated from circFAM188B; this was designated circFAM188B-103aa. Based on previous reports, we performed LC-MS/MS analysis on the SMSCs overexpressing circFAM188B to confirm the detection of circFAM188B-103aa. In this analysis, two peptides matching circFAM188B-103aa were detected. Unfortunately, the two unique amino acids were not detected. Taken together, we suggest that the circular RNA circFAM188B encodes a novel protein, circFAM188B-103aa.

Previous studies have shown that circRNA-translated proteins have unique functions. For instance, circFBXW7-185aa, which is encoded by circular RNA circFBXW7, represses glioma tumorigenesis (Yang et al., 2017); circRNA circPPP1R12A encodes a 73-aa small peptide, circPPP1R12A-73aa, which promotes the growth and metastasis of colon cancer cells (Zheng et al., 2019). In our study, we found that circFAM188B-103aa overexpression promotes proliferation, but inhibits differentiation of chicken SMSCs, which consistent with the function of its parental transcript, circFAM188B. The translated product of circFAM188B shares most of its aa sequence with that of FAM188B, with the exception of two unique amino acids in the ORF located at the C-terminus. However, this difference of two amino acids is insufficient to allow preparation of the antibodies required to gain clarify the underlying mechanisms by which circFAM188B-103aa regulates skeletal muscle development in chicken.

## Conclusion

**FIGURE 9:**
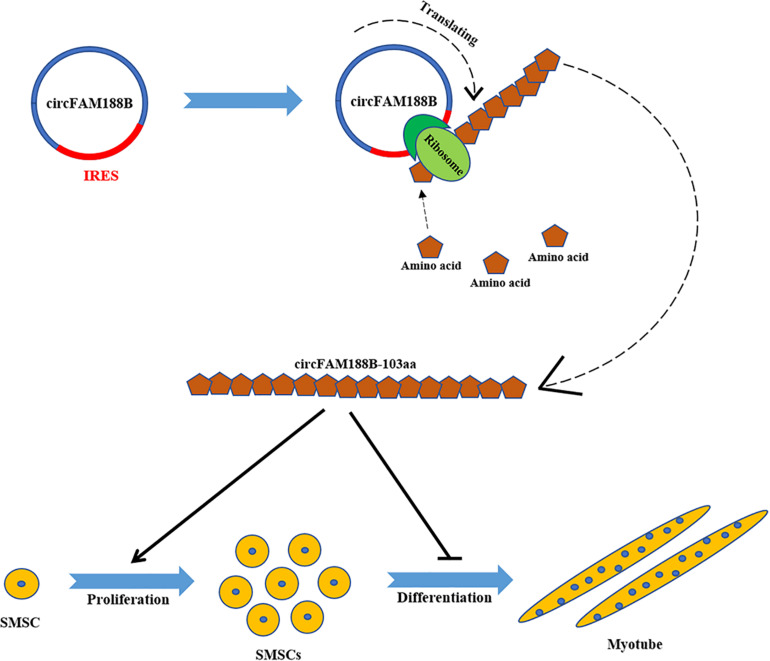
A schematic model of CircFAM188B regulates chicken SMSC proliferation and differentiation. IRES drives a novel circular RNA circFAM188B translation into protein circFAM188B-103aa to promotes the proliferation and inhibits the differentiation of chicken SMSCs.

In conclusion, we have identified a novel circular RNA circFAM188B, which encodes a novel protein circFAM188B-103aa to promotes proliferation and inhibits differentiation in chicken SMSCs (Figure 9). Overall, our findings provide a novel insight into the role of circRNAs in the regulation of skeletal muscle development.

## Data Availability Statement

The raw data supporting the conclusions of this article will be made available by the authors, without undue reservation, to any qualified researcher.

## Ethics Statement

The animal study was reviewed and approved by the Animal Care and Use Committee of Sichuan Agricultural University. Written informed consent was obtained from the owners for the participation of their animals in this study.

## Author Contributions

HY, XS, and QZ: conceptualization. HY, XS, JZ, and XC: formal analysis. HY and QZ: funding acquisition and writing – review and editing. XS, JZ, XC, SH, and YHW: investigation. HH: methodology. YW: project administration. DL: software. YC and CC: validation. HY and XS: writing – original draft. All authors contributed to the article and approved the submitted version.

## Conflict of Interest

The authors declare that the research was conducted in the absence of any commercial or financial relationships that could be construed as a potential conflict of interest.
