# Supplementary material for: Circular RNA CircFAM188B Encodes a Protein That Regulates Proliferation and Differentiation of Chicken Skeletal Muscle Satellite Cells
Source: Front Cell Dev Biol. 2020 Nov 6;8:522588. doi: 10.3389/fcell.2020.522588 (PMC7677141; doi:10.3389/fcell.2020.522588)
Supplement: Supplementary file 2 [file Data_Sheet_2.docx]

**Circular RNA circFAM188B encodes a protein that regulates proliferation and differentiation of chicken skeletal muscle satellite cells**

**Huadong Yin^#^, Xiaoxu Shen^#^, Jing zhao^#^, Xinao Cao^#^, Haorong He, Shunshun Han, Yuqi Chen, Can Cui, Yan Wang, Diyan Li and Qing Zhu ^*^**

*Farm Animal Genetic Resources Exploration and Innovation Key Laboratory of Sichuan Province, Sichuan Agricultural University, Chengdu, Sichuan 611130, China*

^#^ These authors contributed equally to this work.

^*^ Corresponding author:

Qing Zhu, Farm Animal Genetic Resources Exploration and Innovation Key Laboratory of Sichuan Province, Sichuan Agricultural University, Chengdu, Sichuan 611130, China. E-mail: zhuqing@sicau.edu.cn

Supplementary Figure


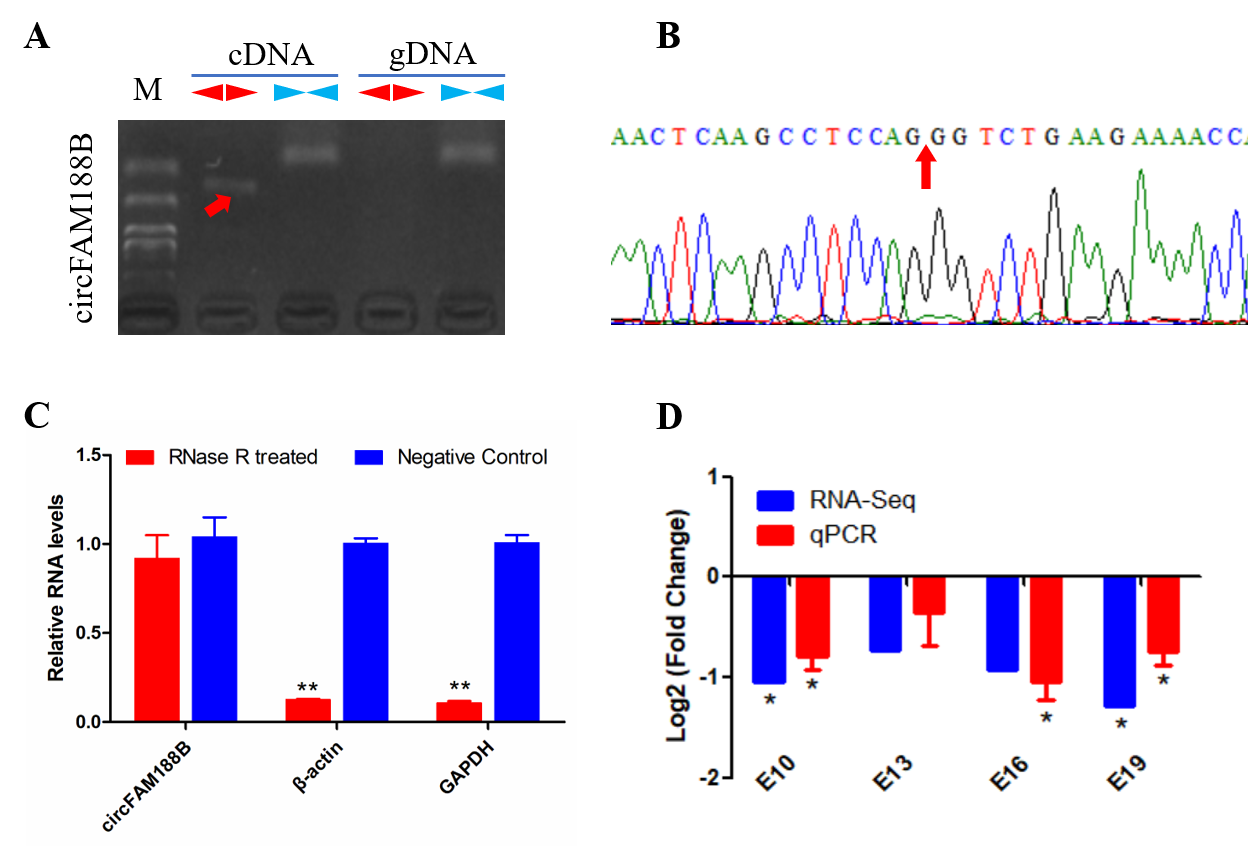


**Figure S1. Experimental validation of circFAM188B. (A)** Divergent primers and convergent primers amplify results of circFAM188B in cDNA and gDNA samples, Divergent primers from circFAM188B produced a single distinct band only in cDNA samples. **(B)** Sanger sequencing confirmed the back-splicing junction sequence of circFAM188B (Red arrow points to the splicing site). **(C)** qRT-PCR results showed that the circFAM188B were more resistant to RNase R than GAPDH and β-actin mRNA. **(D)** qRT-PCR validation indicated that circFAM188B expressed at lower levels in broilers than in layers at embryonic day 10 (E10), E16 and E19.


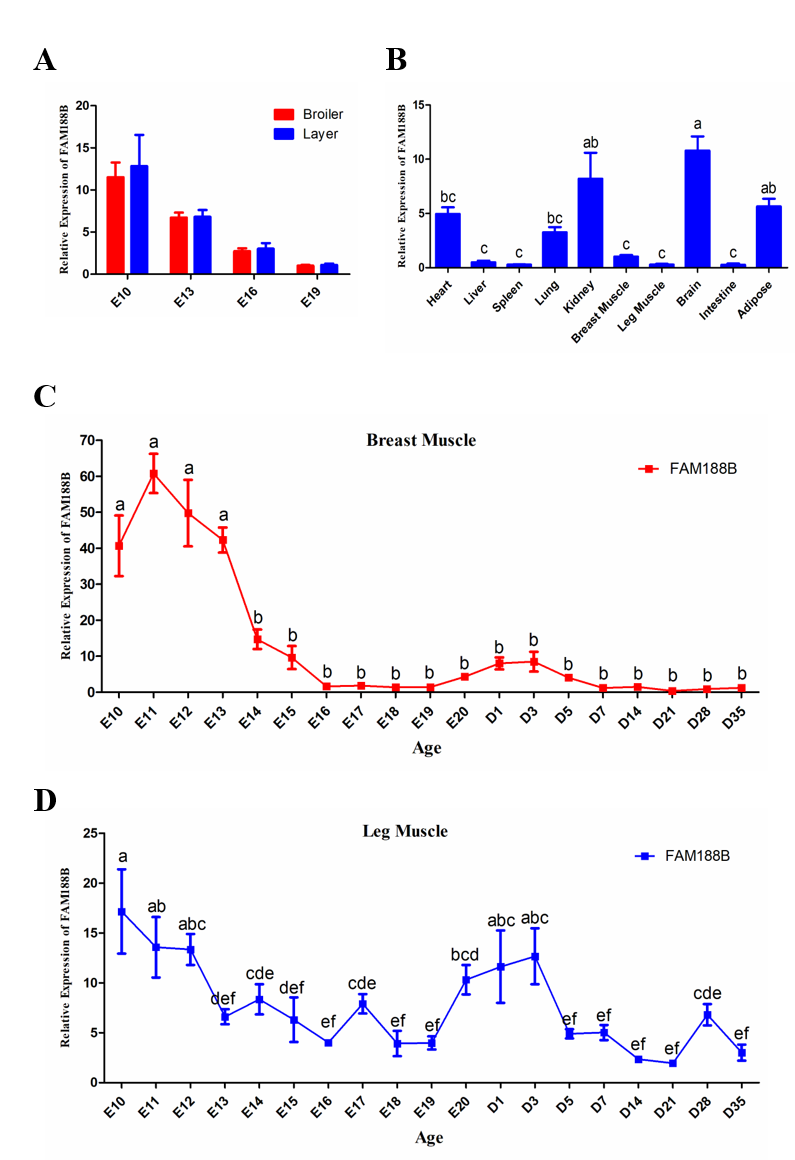


**Figure S2. FAM188B mRNA expression patterns in chicken. (A)** FAM188B expression in breast muscles of four comparison groups (broiler VS. layer). **(B)** circFAM188B expression profiles in ROSS-308 chicken tissues. **(C)** The relative RNA level of FAM188B in the breast muscles of E10-D35 chicken. **(D)** The relative RNA level of FAM188B in the leg muscles of E10-D35 chicken.


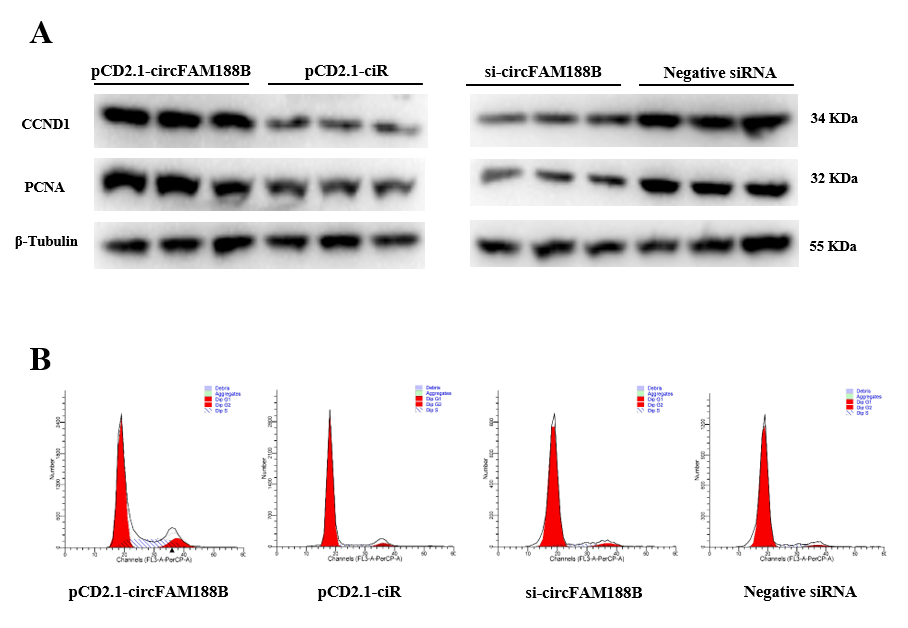


**Figure S3.** **(A)** The protein level of CCND1, PCNA and β-tubulin were analyzed by western blot in SMSCs after circFAM188B overexpression or inhibition. **(B)** cell cycle analyzes of SMSCs after circFAM188B overexpression or inhibition.


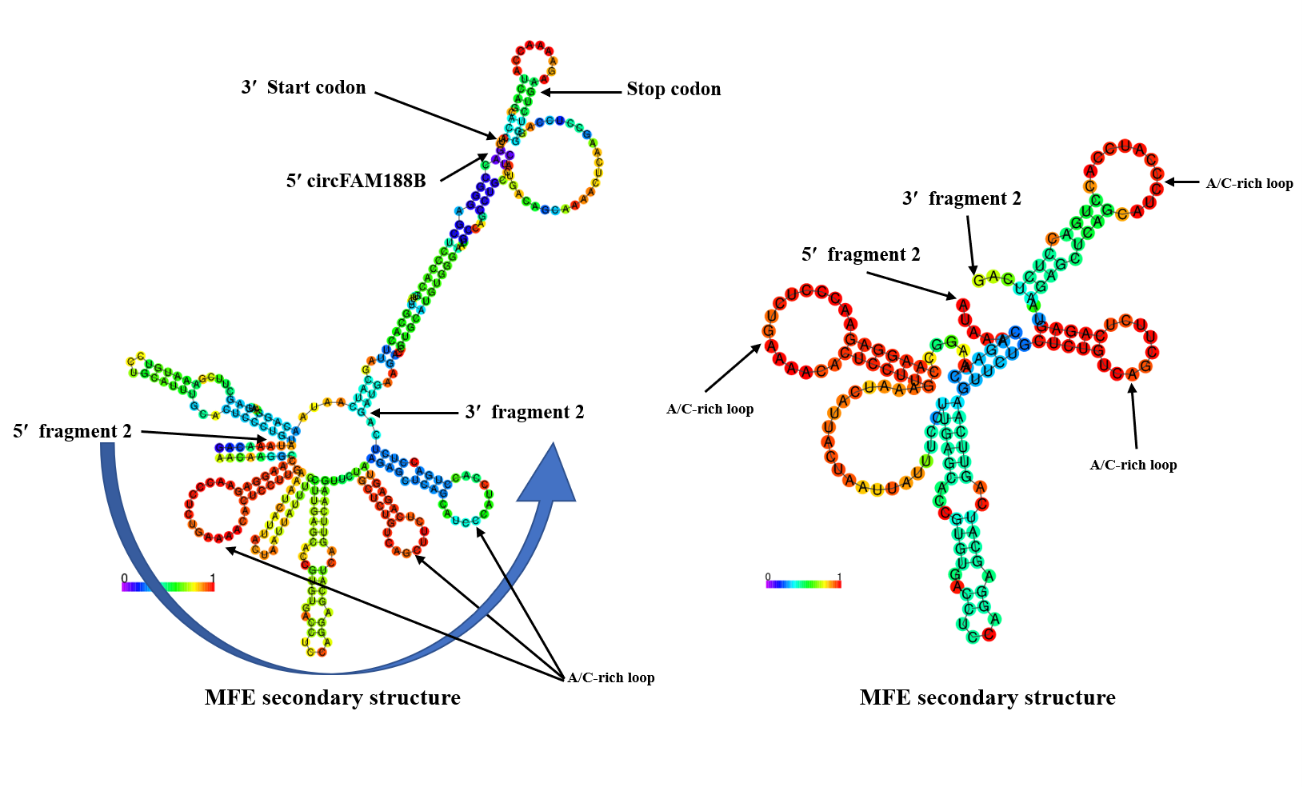


**Figure S4. the RNA secondary structure analysis of circFAM188B.** The linear sequence of circFAM188B were divide into 3 fragments according to the secondary structure of RNA, the fragment 2 was the most complicated one and contain 3 A/C-rich loops.


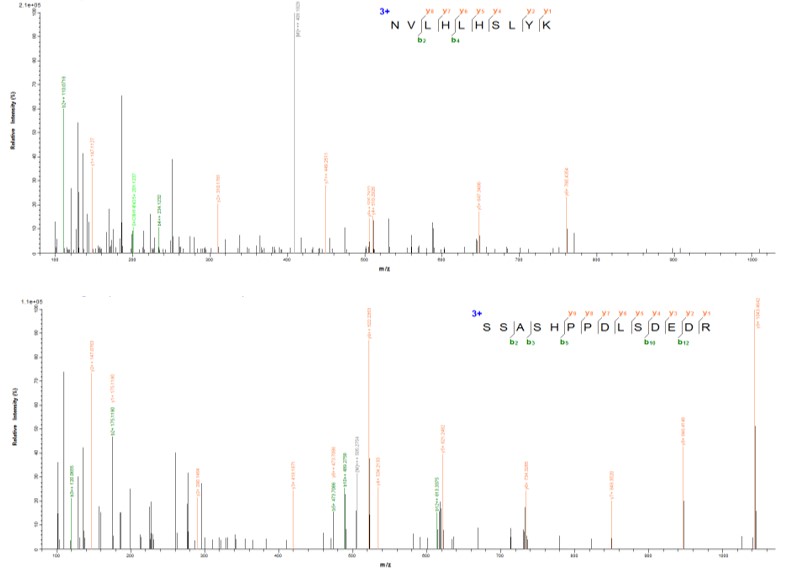


**Figure S5.** the specific charge of corresponding peptide of the FLAG-tagged circFAM188B-103aa identified by LC-MS/MS analysis.


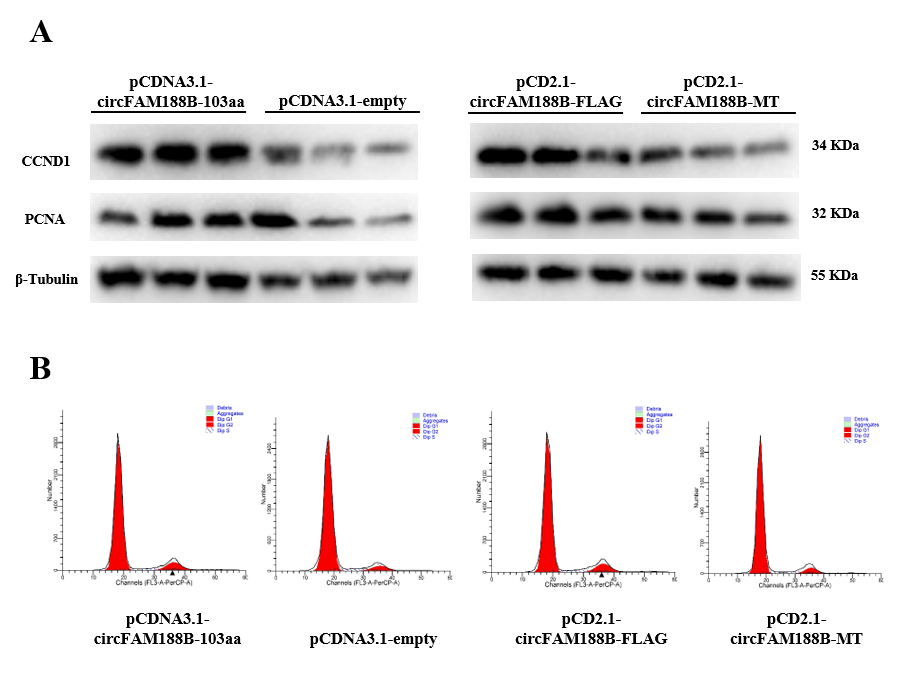


**Figure S6. (A)** The protein level of CCND1, PCNA and β-tubulin were analyzed by western blot in SMSCs after circFAM188B-103aa overexpression. **(B)** cell cycle analyzes of SMSCs after circFAM188B-103aa overexpression.
